# Beyond constructs and principles: addressing gender-related barriers to high, equitable immunization coverage

**DOI:** 10.3389/fgwh.2024.1367590

**Published:** 2024-04-03

**Authors:** Willow Gerber, Rebecca Fields, Neide Guesela, Khadijah A. Ibrahim Nuhu, Eugene Manika

**Affiliations:** ^1^ MOMENTUM Routine Immunization Transformation and Equity Project, JSI Research and Training Institute, Arlington, VA, United States; ^2^ MOMENTUM Routine Immunization Transformation and Equity Project, JSI, Arlington, VA, United States; ^3^ MOMENTUM Routine Immunization Transformation and Equity Project, JSI Research and Training Institute, Maputo, Mozambique; ^4^ MOMENTUM Routine Immunization Transformation and Equity Project, JSI Research and Training Institute, Abuja, Nigeria; ^5^ MOMENTUM Routine Immunization Transformation and Equity Project, PATH, Kinshasa, Democratic Republic of Congo

**Keywords:** gender, immunization, equity, strategy, capacity-building, vaccination

## Abstract

The global immunization community has only recently recognized that addressing gender-related barriers to vaccination is critical to improving equity and increasing protection against vaccine-preventable diseases. USAID's MOMENTUM Routine Immunization Transformation and Equity project aims to strengthen routine immunization programs to overcome entrenched obstacles to reaching zero-dose and under-immunized children while supporting the introduction of other new vaccines given over the life course. From the outset, the project recognized the need to mainstream gender into its global and country level work, incorporating gender considerations into all phases of the program cycle, from assessment to activity design, strategic communications, monitoring, evaluation, and continuous learning. Its gender mainstreaming efforts focus on five areas of improvement for immunization: service access and convenience; service quality and experience; communication and demand generation for immunization among caregivers (both women and men) and families; making services more responsive to agency and autonomy constraints of female caregivers; and the conditions and circumstances of health workers, who are mostly women. The authors describe approaches the project has applied to build capacity of its own global and country level staff to both recognize the gender dimensions inherent in common obstacles to immunization and ways to address them. Authors describe project activities carried out at global and country levels and share experience and challenges encountered in increasing recognition of gender barriers, moving from theory to practical action in addressing them, building capacity, and gauging the success of the work to date. The lessons learned are useful to colleagues working within the circumstances of time-limited and geography-specific projects whose main focus is to improve equity in immunization.

## Introduction and background

For decades, the health sector has recognized the impact of gender on health outcomes. While reproductive health and family planning, HIV/AIDS, and maternal and child health efforts have been mainstreaming gender for decades, the global immunization community has only recently recognized that gender-related barriers lie on a critical pathway to achieving high and equitable vaccination coverage (1). With the need to assure gender equality now reflected in the core principles of both the Immunization Agenda 2030 (2) and the 2021–2025 strategic plan of Gavi, the Vaccine Alliance (3), momentum is increasing to reduce gender barriers that restrict the access to, demand for, and utilization of immunization services that provide protection against a growing number of vaccine-preventable diseases.

This paper shares strategies and experience with addressing gender-related barriers to immunization from the MOMENTUM Routine Immunization Transformation and Equity project (the project), the United States Agency for International Development (USAID) global technical leadership project for immunization. Supporting 20 countries globally^1^ during the period of 2020–2026, the project aims to strengthen routine immunization (RI) to overcome entrenched obstacles to reaching zero-dose and under-immunized children while supporting the introduction of COVID-19 and other new vaccines given over the life course. Central to the project's approach is identifying and addressing the root causes of impediments to equitable vaccination coverage across the life course, including those related to gender.

## Setting forth a vision and practical strategy

In its first year, the project developed a gender strategy that builds on USAID's 50-year history of addressing gender equality and women's empowerment, work that has generated a substantial knowledge base on gender integration grounded primarily in reproductive and maternal health. The Agency's ongoing commitment to reduce gender-related barriers across all 14 of its development sectors is reflected in the USAID Gender Equality and Female Empowerment Policy (4).

**Figure 1:**
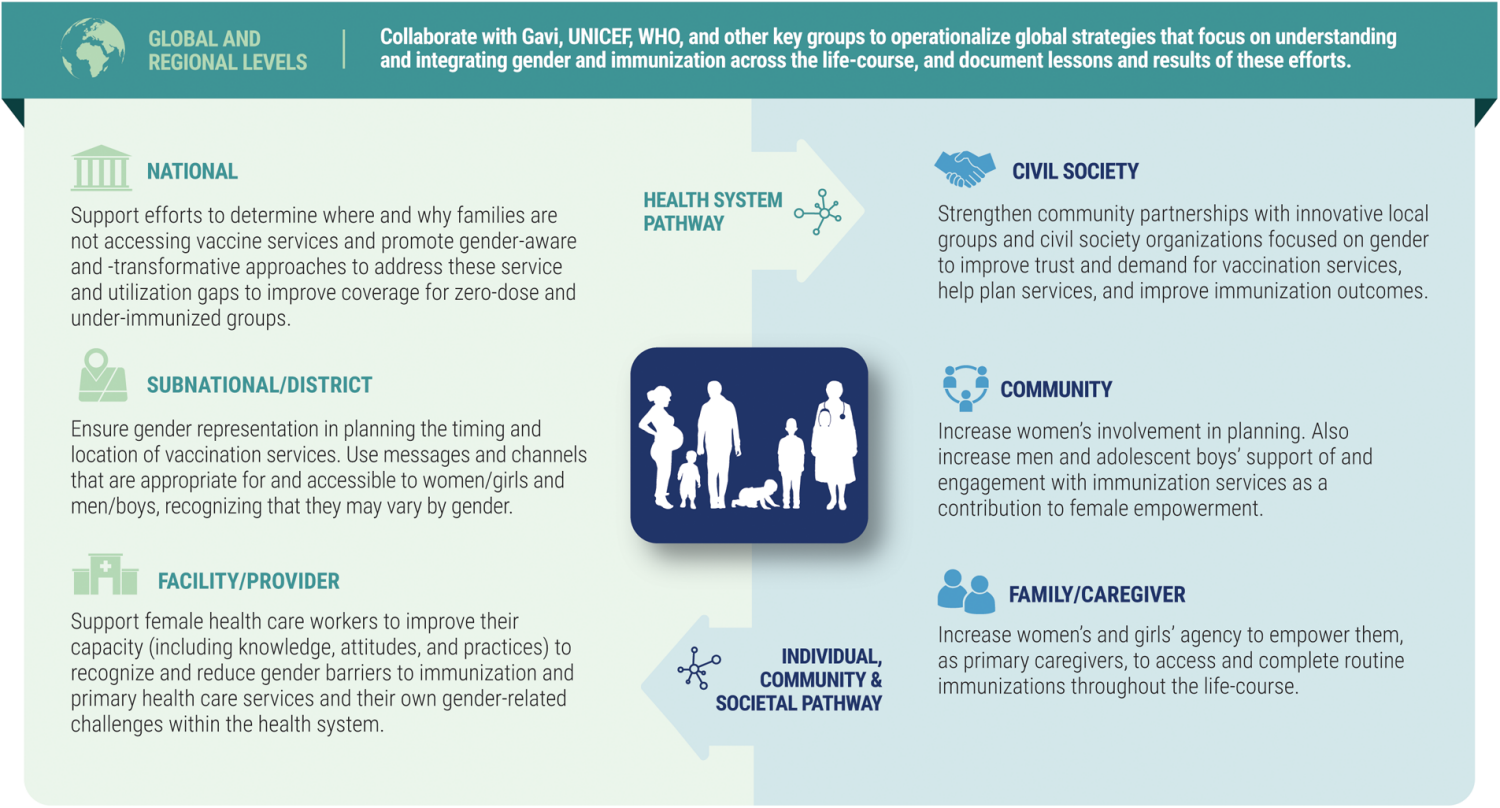
Key elements of MOMENTUM routine immunization transformation and equity gender strategy.

The project's own gender strategy reflects its focus on immunization. As shown in Figure 1, it emphasizes the need to work concurrently across the health system and civil society pathways at multiple levels, from households and health centers all the way up to national programs. It also recognizes that collaboration at global, regional, and country levels is vital to developing synergies: to heighten recognition of the importance of gender in immunization; share tools, experience, and learning; strategically plan and exploit opportunities for coordinated action; and make gender integration for immunization more transparent and practical for all stakeholders in order to achieve better immunization outcomes.

The project mainstreams gender into its global and country level work, incorporating gender-intentional considerations into all phases of its program cycle, from assessment to activity design, strategic communications, and monitoring, evaluation, and continuous learning.

To operationalize the strategy, the project's senior gender advisor organized a series of orientations for staff at global and country program levels in English, French, and Portuguese and established an internal working group of staff from different project teams and country programs that meets biweekly. The group champions gender integration and helps to monitor and provide input for gender-related activities, serving both as a technical learning hub and a supportive community of practice (COP) to elevate the capacity for gender programming for the whole project. The project also identified gender focal points across key country programs to help ensure that gender and equity considerations are recognized and prioritized during workplanning, activity design, implementation, and monitoring, evaluation, and learning.

However, conceptual challenges have persisted in putting the strategy into practice and aligning it with the project's desired outcomes for immunization. To increase awareness and understanding of gender's practical relevance to the project's overall goal of improving equity in immunization, technical staff outlined five strategic areas for gender-related work that are within the project's managerial scope, namely:

1. Improving **access and convenience** of immunization services so that caregivers (who are mostly women) taking children for vaccination can access them (1).
2. Improving **communication and demand generation for immunization with caregivers (both women and men) and families**, focusing on the importance and safety of vaccination, the vaccination schedule, and where and when to go for vaccination.
3. Improving the **technical quality and experience of immunization** so that it is safe, effective and a positive experience that invites completion of the vaccination schedule.
4. Improving the **agency and autonomy of caregivers** (who are generally women) to be able to make use of the services (1).
5. Improving the **conditions and working circumstances of health workers**, who are mostly women (5).

These strategies are compatible with the project's overall approach to overcoming entrenched obstacles in immunization through the active use of human centered design (HCD) and co-creation methods that center on the perceptions, needs, and circumstances of a range of stakeholders.

## Moving from assessment to action

In each country where the project works on RI, it conducts an initial assessment that employs a mix of quantitative and qualitative methods to understand the assets and gaps in immunization programs. These assessments review policies and strategies, program performance, service quality, and perceptions of vaccines and vaccination. While not focused specifically on gender, they use a gender lens to identify gender-related barriers. Given the newness of attention to gender in immunization, some country teams have been more systematic than others in this effort but the assessments yielded important findings related to gender that were the subject of subsequent attention. A summation of M-RITE's assessment and co-creation work in Democratic Republic of Congo, Mozambique, and Nigeria (6) describes the project's approach of carrying out rapid research (primarily qualitative) followed by gender-sensitive co-creation workshops with caregivers, community members, health workers, and other health system actors. This approach convened a range of stakeholders to jointly identify context-appropriate, feasible solutions to help address root causes of barriers to equitable RI coverage, several of which are gender related. These locally designed solutions informed the project's country workplans, ultimately translating findings into action.

The following brief descriptions illustrate the range of the Project's country-level gender integration activities to advance more equitable immunization outcomes.

## Democratic Republic of Congo: improving women's health literacy and demand for vaccination

In **DRC**, the project collaborates with a local women's rights organization, Femmes Mains dans la Main pour le Développement Intégrale (FMMDI) in the Sanga Nyembwe health area in Kasaï-Central Province to improve the use of vaccination services. In this location, more than 20,000 inhabitants are dispersed across a large geographic area that has only one vaccination site and low levels of female education and literacy, circumstances that limit both access to and demand for vaccination services. With project support, FMMDI engages members of the Kasaï-Central Provincial Health Department, civil society groups including women's and youth organizations, political and administrative authorities, and local businesspeople to co-create local solutions through the use of HCD methods. Solutions include raising awareness among grassroots women's organizations on the importance of vaccinating children and women of childbearing age and mobilizing resources to increase demand for vaccination. FMMDI has incorporated information on vaccination into the instructional materials it uses with women who are learning to read and write. These women have themselves become peer educators for other women in the community, creating a multiplier effect focused on awareness-raising and referrals to the health center. The continuous focus on facilitating women's use of vaccination services has led to substantial increases in the numbers of doses of vaccines administered to both children and women of child-bearing age.

## Mozambique: expanding community and male engagement in immunization

Around the world, women are generally held responsible for their children's health, including RI (7, 8). In **Mozambique**, the project works to improve men and boy's understanding of RI and what they can do to support their families' well-being. The Project used gender-sensitive assessment methods to identify entrenched obstacles to RI and underlying causes in districts in Nampula and Zambezia provinces. These findings were used to develop activities that include working closely with community health committees, community health workers, and religious leaders. One aim is to increase their recognition of the importance of male engagement in RI, reduce the imbalance of power between mothers and fathers, and build capability to negotiate ways to meet their children's immunization and health needs. Adapting existing resources such as “*Do's and Don'ts of Engaging Men and Boys*” (9) to RI, the project conducts capacity building for health workers and community dialogues with community members to discuss practical actions that men and boys can take to help increase child immunization. These discussions highlight their role as clients, partners, and change agents for immunization and the importance of a supportive family structure for children's overall well-being. The vaccination calendar is used as a key instrument for equipping community focal points and female and male caregivers with essential information on when to come for vaccination and the vaccines to be provided. Project efforts are tailored to the specific social norms and educational levels of different communities. This work is amplified through working with local language community radio stations to discuss community support for and male engagement in RI without distracting attention from women's needs and agency in this area.

## Nigeria: building gender considerations into supportive supervision to improve service quality

In **Nigeria**, the country with the highest number of zero-dose children in Africa (10), the project provides technical support to the National Primary Health Care Development Agency and the states of Bayelsa, Edo, Imo, Jigawa, and Lagos. The Project's gender integration efforts focus primarily on two areas, community engagement and service quality. For the former, the project helped review the National Community Engagement Strategy and, at the state level, provides technical assistance for the updated communication and demand generation strategy, and builds capacity to address social and gender norms through engagement with community mobilizers, nurturing a foundation for gender transformative programming. For the latter, the project works with local immunization officers to introduce a gender equity lens into their supportive supervision activities. Working from an existing framework on gender transformative supervision (11) and adapting it for RI purposes, the team is assessing the perceived sensitivity of immunization officers in Imo state to gender-related barriers in their respective settings. In this nascent effort, the project is also supporting the state team in Edo where the officers are gaining more awareness and insight into the gendered dynamics of supportive supervision and are now part of a growing number of gender-aware and -responsive stakeholders working to test the introduction of gender-sensitive supportive supervision elements into their states' RI supportive supervision checklists.

## Growing a cadre of capable practitioners for gender and immunization

Beyond M-RITE's commitment to building the capacity of its own staff and local partners to advance gender in immunization capacity, commitment, and confidence, the project aimed to build a wider cadre of advocates and practitioners. In 2022 it conducted a four-session, month-long online short course titled *Gender in Immunization: Opportunities for Action*, offered through The Sabin Vaccine Institute's BOOST platform. Initially offered in English and focused on national and subnational participants from lower- and middle-income countries (LMICs), approximately 130 professionals from over 30 countries joined the interactive program, with 100 meeting the stringent requirements to receive a certificate of completion. Collaborative alliances with gender experts at Gavi, UNICEF, and WHO ensured that the most current knowledge and tools were included and that well-recognized speakers shared their expertise. Course facilitators prioritized peer exchanges, featuring country-level implementers who presented on their work and lessons learned. The course made use of Gavi-funded short learning videos released just in time for the event (12–14). Facilitators made a point of introducing and actively using key tools and materials on gender developed by WHO (15), UNICEF (16–18), and Gavi (19).

In response to demand for a French version, the project updated the course for francophone LMICs with adapted materials and content. For this effort, the project worked closely with a civil society organization active in West and Central Africa, the Organisation d'Afrique Francophone pour le Renforcement des Systèmes de Santé et de la Vaccination (*OAFRESS*), which provides technical assistance to over 1,500 members across 18 Francophone countries. OAFRESS staff and members were featured as speakers and shared experiences and learning from their countries. Over 100 participants from 14 countries earned certificates with several requesting the project to provide further opportunities for learning and peer exchange. In response, the project will launch a francophone COP in the upcoming program year.

## Continuing challenges (discussion)

In implementing the activities described above, the project has seen both advances and continuing challenges in addressing gender-related barriers. There is now greater recognition of the role that gender plays in immunization and of the gendered dimensions to common problems in vaccination. Examples include vaccine stockouts that necessitate additional visits to health facilities and costs incurred to caregivers; inconvenient times and places for vaccination; and inadequate counseling on the vaccination schedule and how to manage common side effects of vaccines, which can lead to conflict between male and female partners about the need for the child to complete the vaccination schedule.

Important challenges persist. Project staff report that some health officials do not view gender as a legitimate issue that merits attention, instead focusing on more traditional approaches to problem-solving that do not reference dimensions that challenge social or cultural norms. There is also a widespread perception that while assessments can identify gender-related barriers, it is not possible to respond to them with manageable, evidence-based solutions that yield change in the near to medium term. New toolkits and resources are being rolled out and major capacity-building efforts to build proficiency in gender-intentional programming for immunization are underway with support from Gavi (20, 21). However, a gap remains in going from training and assessment to action that addresses gender-related barriers as a standard element of immunization programming.

In some cases, this is due to the lack of recognition of the gender dimensions of performance improvement efforts, such as expanding the times and locations for vaccination services or including mothers in immunization microplanning or feedback sessions. Another key need expressed by project staff is for user-friendly measures and methods that can be used by local health officials to monitor whether efforts to improve gender equality in immunization are working. The project is currently developing and field-testing indicators and methods that are directly relevant to program actions and do not require expensive and infrequent household surveys, recognizing that it is unlikely that there will be a direct, linear relationship between gender-related activities and vaccination coverage. Instead, triangulation across indicators will be needed. These project efforts complement actions by global immunization partners to introduce gender indicators for the annual WHO/UNICEF joint reporting form.

Much work remains to be done to mainstream gender considerations into initiatives to achieve high and equitable immunization coverage not just for children but over the life course. The project will continue to build capacity, implement, assess, and document activities to support national and subnational governments and civil society organizations in achieving high and equitable coverage. It will continue to collaborate with global immunization partners to increase the commitment, capability, and confidence of health practitioners and civil society at all levels to achieve high and equitable protection against all vaccine-preventable diseases.

## Data Availability

The original contributions presented in the study are included in the article/Supplementary Material, further inquiries can be directed to the corresponding author.

## Footnotes

^1^Benin; Bosnia Herzegovina: Côte d’Ivoire; Ethiopia (ended); Bosnia & Herzegovina; Serbia; N. Macedonia; Moldova; Ghana (ended); Haiti; India; Kenya; Kyrgyz Republic (ended); Mali (ended); Mozambique; Niger; Nigeria; Serbia; South Sudan (ended); Vietnam (ended).
